# Association between processed red meat intake and cardiovascular risk factors in patients with type 2 diabetes: a cross-sectional study from China

**DOI:** 10.3389/fnut.2024.1438953

**Published:** 2024-08-29

**Authors:** Fan Zhang, Yongfei Chai, Jiajia Ren, Xiaoyu Xu, Cuiqi Jing, Haimeng Zhang, Yuhong Jiang, Hong Xie

**Affiliations:** ^1^School of Public Health, Bengbu Medical University, Bengbu, China; ^2^Qilin Community Health Service Center, Nanjing, China

**Keywords:** processed red meat, type 2 diabetes mellitus, cardiovascular disease, cross-sectional survey, risk factors

## Abstract

**Aim:**

To explore the association between Processed red meat (PRM) consumption and cardiovascular risk factors in Chinese adults with type 2 diabetes mellitus (T2DM).

**Methods:**

Dietary survey, physical measurement, and blood biochemical examination were conducted on 316 patients with type 2 diabetes in Bengbu, China from May to July 2019. Possible confounding factors were identified by comparing between-group variability in the baseline table. To eliminate the effect of confounding factors, subgroup analysis was used to explore whether there were differences in the correlation between PRM intake status and the indicators in cardiovascular disease risk factors. A logistic regression model was used to analyze the association between PRM and the risk of abnormal levels of cardiovascular risk factors in T2DM patients. Restricted cubic spline plots were used to analyze the dose–response relationship between PRM intake and the indicators of cardiovascular disease risk factors.

**Results:**

A total of 316 subjects were included in the study, of whom 139 (44.0%) were male and 177 (56.0%) were female. In the multiplicative interaction, there was an effect modifier for smoking (Pinteraction = 0.033) on the association between PRM intake and the risk of substandard FPG level control; sex (Pinteraction = 0.035), smoking status (Pinteraction = 0.017), and alcohol consumption (Pinteraction = 0.046) had effect modifying effects on the association between PRM intake and risk of abnormal systolic blood pressure. Sex (Pinteraction = 0.045) had an effect modifier on the association of PRM intake status with the risk of diastolic blood pressure abnormality. In addition, age had an effect modifier on the association of PRM intake status with risk of abnormal triglyceride index (Pinteraction = 0.004) and risk of abnormal HDL index (Pinteraction = 0.018). After adjusting for potential confounding variables, logistic regression showed that the OR for substandard HbA1c control in patients in the highest PRM intake group, T3 (3.4 ~ 57.2 g/d), was 1.620-fold higher than in the lowest intake, i.e., the no-intake group, T1 (0.0 ~ 0.0 g/d; OR = 2.620; 95% CI 1.198 ~ 5.732; *p* = 0.016). Whereas the OR for abnormal control of systolic blood pressure levels was 1.025 times higher (OR = 2.025; 95% CI 1.033 ~ 3.968; *p* = 0.040) in patients in the PRM low intake group T2 (0.1 ~ 3.3 g/d) than in the non-intake group T1 (0.0 ~ 0.0 g/d), the OR for substandard control of systolic blood pressure in patients in the highest group T3 (3.4 ~ 57.2 g/d) was 1.166 times higher than in the no-intake group T1 (OR = 2.166; 95% CI 1.007 ~ 4.660; *p* = 0.048). The OR for abnormal TG levels in patients in the highest PRM intake group T3 (3.4 ~ 57.2 g/d) was 1.095 times higher than in the no-intake group T1 (OR = 2.095; 95% CI 1.076 ~ 4.078; *p* = 0.030). Restricted cubic spline plots presented a nonlinear dose–response relationship between PRM intake and risk of substandard HbA1c and SBP control (P nonlinear <0.05), and an atypical inverted U-shaped association between PRM intake and TC and LDL-C levels (P nonlinear <0.05). The strength of the associations between PRM intake and the control levels of FPG, DBP, HDL-C, and TG were not statistically significant (*p* > 0.05).

**Conclusion:**

PRM intake was generally low in patients with T2DM, but a nonlinear dose–response relationship between PRM intake and the risk of suboptimal control of HbA1c and SBP, with an atypical inverted U-shaped association with TC and LDL-C levels, was observed. Appropriate control of PRM intake may be important for tertiary prevention of T2DM and cardiovascular disease prevention. We need to better understand these relationships to promote improved cardiometabolism and global health.

## 1 Introduction

Type 2 diabetes mellitus (T2DM) refers to a type of diabetes mellitus with insulin resistance with progressive insulin secretion insufficiency as the main etiology (1). T2DM has become one of the key problems of global public health because of the large number of patients, the low control rate of blood pressure, blood lipids, and glucose metabolism, the difficulty in the control of cardiovascular diseases and other complications, and is an accelerating public health challenge (2). From 1980 to 2021, the number of adults with diabetes in the world is estimated to have increased from 108 million to 540 million, and the prevalence rate has reached 10.5%. The International Diabetes Federation estimates that the global prevalence of diabetes will increase sharply in the future, rising to 12.2% (about 780 million people) by 2045, of which T2DM accounts for more than 90% (3). A large part of the diabetes health burden can be attributed to large blood vessels and microvascular complications of diabetes-related. Atherosclerosis sex refers to coronary heart disease, cardiovascular disease cerebrovascular disease, or peripheral artery disease, is considered to be the origin of atherosclerosis, and is a major cause of diabetic complications and death (4). It occurs about 15 years earlier than people without diabetes, and adults with diabetes have a two-to-four-fold increased risk of cardiovascular disease (CVD) compared to adults without diabetes, and the risk increases as glycemic control worsens (5, 6). Contains 57 articles recently published in the systematic review of 4,549,481 patients with T2DM (7), according to the result of CVD deaths accounted for 50.3% of all deaths of T2DM subjects (95% CI: 37.0 63.7%). The above situation reminds us that such a fact must make great efforts to improve the risk of diabetes and cardiovascular disease (8, 9).

Lifestyle modification, especially dietary modification, is a widely accepted cornerstone method for the prevention of T2DM and CVD and is the first-line strategy for the prevention of T2DM and CVD (10). The analysis of the Global Burden of Disease Study 2019 suggested that 44% of the burden of diabetes was attributed to dietary factors, among which two of the top three factors were high intake of red meat and PRM (11). Excessive intake of saturated fatty acids can significantly increase the concentration of plasma low-density lipoprotein cholesterol, thereby increasing the risk of CVD (12) and coronary heart disease (13). Reducing the intake of saturated fatty acids can reduce CVD events. However, the results of recent studies on the association of PRM consumption with CVD and diabetes remain inconsistent and largely uncertain. Research thought, that unprocessed and processed red meat consumption are linked to cardiovascular disease, cardiovascular disease diabetes is associated with high-risk subtypes and (14). Another Mendelian randomization study found no significant effect of red meat and PRM consumption on coronary artery disease, hypertension, stroke, and T2DM (15). A microsimulation study in the United States concluded that reducing PRM consumption could reduce the burden of some chronic diseases in the United States, including diabetes and CVD (16). The results of an umbrella systematic review suggest (17) that red and processed meat intake may not be causologically related to cardiovascular disease, but may be causologically related to T2DM. More studies are still needed to explore.

In addition, most of the studies on the association between PRM and diabetes and cardiovascular disease were based on healthy populations. Few studies have analyzed the effect of PRM intake on glycemic control in T2DM patients and the effect of PRM intake on CVD in T2DM patients. Diabetes mellitus patient population compared with healthy people, a lot of changes in the environment, such as sugar, lipid metabolic disorder, and insulin resistance, such as internal adjustment ability is abated, more sensitive to dietary factors, which in turn affect the reaction. In addition, the dietary guidelines for diabetes recommend that patients with T2DM “moderate livestock meat intake and limit PRM intake,” but there is a lack of recommended intake. This study aimed to describe PRM intake and evaluate the relationship between PRM intake and the risk of abnormal control of cardiovascular risk factors in patients with T2DM in the Chinese community, and to provide dietary reference data for tertiary prevention, CVD prevention, and primary care of T2DM patients in the community.

## 2 Methods

### 2.1 Study design

The data used in this study were collected from a community-based disease profile of the ‘Provincial Chronic Disease Prevention and Control Demonstration Area’ project, which was initiated in May–July 2019 in Bengbu City, China. All T2DM patients (those who had been diagnosed with diabetes in the hospital before the survey) within the jurisdiction of a community health service station in Bengbu City were selected as survey respondents by cluster sampling method. Inclusion criteria: patients with T2DM aged 18 years and older who had lived in the area for more than 6 months in the past 12 months. Exclusion criteria: (1) those with a history of psychiatric disorders and cognitive impairment; (2) those with severe organic diseases and mobility problems; (3) pregnant and lactating women. This study was a cross-sectional study, based on the study of Wang Limin et al. (18), which set the diabetes control rate of 49.2% as the expected presenting rate (P) in T2DM patients, set the relative permissible error (d) to be no more than 0.15 P, and the significance level α to be taken as 0.05 bilaterally, and used the formula for calculating the sample content of cross-sectional studies, with the calculation process and results as follows: *n* = Z^2^_α/2_P(1-P)/d^2^. Therefore, the effective sample size to be investigated in this study was 176 patients with T2DM, and considering factors such as non-response and questionnaire validity in the actual investigation process, this sample size was appropriately enlarged by 15%, i.e., at least 202 patients were required to be investigated, and 316 cases were recovered at the end as the actual sample size for the investigation. The median intake among the 30.7% of participants who ingested PRM was 3.3 g/d (see Supplementary file), and according to the distribution of their intake, patients with T2DM were divided into three groups: the non-intake group T1 (0.0 ~ 0.0 g/d), the low intake group T2 (0.1 ~ 3.3 g/d) and the high intake group T3 (3.4 ~ 57.2 g/d). This study was approved by the Ethics Committee of Bengbu Medical College (Lunke Approval [2016] No. 15). Informed consent was obtained from the respondents in the survey.

### 2.2 Investigation and measurement

1. Survey: A combination of centralized and household surveys was used to collect socio-demographic information (including sex, age, monthly income, and literacy level) and lifestyle information (including smoking, alcohol consumption, and physical activity level) and medication status of the respondents using a questionnaire developed by the subject team.
2. Anthropometric measurements: Systolic blood pressure (SBP), diastolic blood pressure (DBP), body weight, height, and body mass index (BMI) were collected by trained investigators through standardized procedures and uniform instruments. Body Mass Index (BMI) = calculated as weight (Kg)/[height (m)]^2^, measured in units of (kg/m^2^).
3. Laboratory tests: The participants were required to fast after 8 p.m. the evening before the examination. Blood samples were collected in the early morning of the examination day to detect the concentrations of glycated hemoglobin A1c (HbA1c), fasting plasma glucose (FPG), triglycerides (TG), total cholesterol (TC), high-density lipoprotein-cholesterol (HDL-C) and low-density lipoprotein-cholesterol (LDL-C), measured in mmol/L units. The day before the blood draw, the community physician carried out relevant publicity and mobilization in the community and asked again about the feeding time before drawing blood. The collected blood samples would be sent to the laboratory of the affiliated hospital to have the results tested.

### 2.3 Dietary assessment

The dietary data of T2DM patients over the past year was collected using a validated semi-quantitative food frequency questionnaire (FFQ) developed by Chinese scholars combined with food models (19), including underrepresented minorities (URM), persons with reduced mobility (PRM), sugary beverages, and pure energy foods. Before the survey, to ensure data accuracy, surveyors made multiple visits to the community to promote the study and conducted training simultaneously. Food groups were classified according to the Chinese Food Composition Table (Standard Edition), 6th edition/Volume 1 and Volume 2 (20). The food frequency method is used to calculate the average daily intake of various foods for patients by multiplying the frequency of consumption (times/day, times/week, times/month, or times/year) and the amount consumed each time (g/time or mL/time), which are then converted into a unified unit of “g/d or mL/d.” The average daily intake of various foods (g/d or ml/d) and the daily total energy intake level (kcal/d) are calculated by investigators using the Food Nutrition Calculator V2.65 developed and recommended by the National Institute of Nutrition and Food Safety, Chinese Center for Disease Control and Prevention.

### 2.4 Concept definition

1. T2DM patients: According to the “Chinese Type 2 Diabetes Prevention and Treatment Guidelines (2017)” (21), T2DM is defined as having fasting plasma glucose (FPG) > 7.0 mmol/L, or hemoglobin A1c (HbA1c) > 6.3% at the time of investigation, or having been previously diagnosed by a hospital or using insulin or oral hypoglycemic drugs within 2 weeks.
2. The control rate of diabetes (%) = the number of diabetic patients with FPG < 7.0 mmol/L/the total number of diabetic patients × 100% (22).
3. Red meat (RM) refers to livestock meat including muscles and offal of pigs, cows, sheep, and other domestic animals, which is rich in high-quality proteins, essential fatty acids, vitamins and minerals, etc., and is an important part of a balanced diet (23). According to the different ways of processing RM, it is classified into Unprocessed red meat (URM) and Processed red meat (PRM). Unprocessed red meat refers to fresh, unprocessed red meat; processed red meat refers to red meat that has been treated by baking, smoking, salting, or adding chemical preservatives to extend shelf life or improve flavor (17).
4. Current smokers: defined as those who smoke at least one cigarette per day and have been smoking for at least 1 year (19). Current drinkers: determined based on alcohol consumption in the past year, with no alcohol intake defined as consuming 0 g/d and any amount more significant than 0 g/d defined as current drinking (24).
5. Poor blood glucose control refers to T2DM patients with HbA1c levels ≥7.0% (25). The rate of poor blood glucose control (%) = number of patients with poor blood glucose control/total number of patients ×100%.
6. Physical activity level: expressed in terms of metabolic equivalents (MET), calculated using the Chinese version of the International Physical Activity Questionnaire (IPAQ) to assess different levels of physical activity intensity (26).

### 2.5 Statistical analysis

All statistical analyses were performed using SPSS 25.0, R software (version 4.2.2), and MSTATA software. The distribution of continuous variables was presented as mean ± standard deviation (^−^X ± S) or median (interquartile range), and between-group comparisons were conducted using analysis of variance or the Kruskal-Wallis test. Categorical variables were expressed as rates or proportions, and between-group comparisons were made using the chi-square or Fisher’s exact test. Possible confounders were identified by comparing differences between groups. To eliminate the effect of confounding factors, the association between PRM intake status and the indicators of cardiovascular disease risk factors was investigated by grouping by sex (male and female), smoking status (nonsmoking, smoking), alcohol consumption status (do not drink, drink), and the use of medication (yes and no). The association between PRM intake status and the abnormal level of control of the indicators of cardiovascular disease risk factors in patients with T2DM was analyzed by using a logistic regression model. The relationship between PRM intake and abnormal levels of control of cardiovascular disease risk factors in patients with T2DM was analyzed using a logistic regression model, with model 1 unadjusted for any variable, and model 2 adjusted for age, sex, smoking, alcohol consumption, medication, and total energy intake. In addition, the Akaike information criterion (AIC) (27) was combined to select the optimal model and determine the number of nodes to capture the non-linear relationship and avoid overfitting. Using three segments (HbA1c, SBP, DBP) at the 10th, 50th, and 90th percentiles, four segments (TG) at the 5th, 35th, 65th, and 95th percentiles, and five segments (FPG) at the 5th, 27.5, 72.5 and 95th percentiles, Restricted cubic spline curves with seven segments (TC, HDL-C and LDL-C) at the 2.5, 18.33, 34.17, 50, 65.83, 81.67 and 97.5 percentiles were used to establish the relationship between PRM intake and cardiovascular risk factors. A *p* value of less than 0.05 was considered statistically significant.

## 3 Results

### 3.1 Basic characteristics of the subjects

The basic information of the investigators is shown in Table 1. A total of 316 patients with T2DM aged 18 years and above (mean 65 years) were included in this study, 139 (44.0%) males and 177 (56.0%) females, in which the mean daily intake of PRMs was 2.03 g/d, and only 30.7% of the participants ingested PRMs, and their mean intake was 6.6 g/d (Supplementary Figures 1, 2; Supplementary Table 1). The results showed that for PRM, there was a significant difference in PRM intake between the intake tertiles (*p* < 0.001). The group with the highest PRM intake (T3 group) had a significantly lower age (60 ± 13 years) compared to the other groups (*p* < 0.001). PRM intake was significantly associated with sex (*p* = 0.008) in the different intake groups, and T2DM patients in the T3 group were more likely to be male compared with the PRM T1 group. There was also a significant difference in the use of medication among the different intake groups (*p* = 0.023), with the group with the highest PRM intake being more likely to be T2DM patients treated with medication. Notably, smoking (*p* < 0.001) and alcohol consumption (*p* < 0.001) were observed to exhibit different behavioral patterns at different intake levels. In addition, the total energy intake of the participants was significantly increased (*p* < 0.001) at different levels of PRM intake, with participants in the highest intake group (T3 group) consuming the highest number of calories (2,621 ± 1,005 Kcal).

**Table 1:** Basic information of the study subjects.

	Processed red meat intake (three categories)					
Characteristics	The total number of people, *N* = 316	T1, *N* = 219(0.0 ~ 0.0 g/d)	T2, *N* = 54(0.1 ~ 3.3 g/d)	T3, *N* = 43(3.4 ~ 57.2 g/d)	A statistic	*P* value
**PRM intake (g/d)**	0.00 (0.00, 1.45)	0.00 (0.00, 0.00)	1.70 (1.23, 2.58)	7.10 (5.15, 14.25)	311.49***	**<0.001**^#^
**Age (years)**	65 ± 9	66 ± 8	66 ± 8	60 ± 13	9.36*	**<0.001**^#^
**Sex**					9.63**	**0.008**^#^
Male	139 (44.0%)	95 (43.4%)	17 (31.5%)	27 (62.8%)		
Female	177 (56.0%)	124 (56.6%)	37 (68.5%)	16 (37.2%)		
**Degree of education**						0.759
Primary school and below	62 (19.6%)	45 (20.5%)	12 (22.2%)	5 (11.6%)		
Junior high school	146 (46.2%)	97 (44.3%)	27 (50.0%)	22 (51.2%)		
High school and technical secondary school	82 (25.9%)	57 (26.0%)	12 (22.2%)	13 (30.2%)		
College or above	26 (8.2%)	20 (9.1%)	3 (5.6%)	3 (7.0%)		
**Monthly income (Yuan)**						0.879
<2000	59 (18.7%)	42 (19.2%)	10 (18.5%)	7 (16.3%)		
2000 ~ 3,999	223 (70.6%)	153 (69.9%)	40 (74.1%)	30 (69.8%)		
≥4,000	34 (10.8%)	24 (11.0%)	4 (7.4%)	6 (14.0%)		
**Smoking**					17.62**	**<0.001**^#^
No	254 (80.4%)	180 (82.2%)	49 (90.7%)	25 (58.1%)		
Yes	62 (19.6%)	39 (17.8%)	5 (9.3%)	18 (41.9%)		
**Drinking alcohol**					14.48**	**<0.001**^#^
No	235 (74.4%)	169 (77.2%)	44 (81.5%)	22 (51.2%)		
Yes	81 (25.6%)	50 (22.8%)	10 (18.5%)	21 (48.8%)		
**Take medication**					7.53**	**0.023**^#^
No	82 (25.9%)	47 (21.5%)	19 (35.2%)	16 (37.2%)		
Yes	234 (74.1%)	172 (78.5%)	35 (64.8%)	27 (62.8%)		
**Physical Activity (Metabolic equivalent)**	6,010 ± 2,699	6,075 ± 2,577	6,080 ± 2,454	5,593 ± 3,512	0.59*	0.552
**Body Mass Index (Kg/m^2^**)	25.0 ± 3.5	24.8 ± 3.3	25.2 ± 3.6	25.5 ± 4.1	1.08*	0.339
**Pastry amount (g/d)**	0 (0, 6)	0 (0, 6)	0 (0, 6)	3 (0, 11)	5.12***	0.077
**Total beverage volume (ml/d)**	0.0 (0.0, 0.0)	0.0 (0.0, 0.0)	0.0 (0.0, 0.0)	0.0 (0.0, 0.0)	5.15***	0.076
**Pure energy food (g/d)**	41 (27, 55)	41 (27, 55)	41 (28, 52)	45 (28, 80)	3.50***	0.174
**Total energy Intake (Kcal/ day)**	2,200 ± 892	2,085 ± 845	2,335 ± 881	2,621 ± 1,005	7.53*	**<0.001**^#^

All data are presented as mean ± standard deviation, n (%), median (interquartile range). For comparison of processed red meat(PRM) intake among the three groups, analysis of variance or Kruskal-Wallis test was used for continuous variables, and categorical variables were used χ^2^ or Fisher’s exact test.*Represent F value,**Represent χ^2^ value,***Represent H value. ^#^Represent *p* < 0.05.

### 3.2 Subgroup analysis

Subgroup analyses were performed to eliminate the effects of demographic variables and lifestyle confounders. Subgroups were analyzed by age, sex (male, female), smoking status (non-smoking, smoking), drinking status (non-drinking, drinking), and use of medication (yes, no) to explore the effect of PRM intake status on the risk of abnormal concentrations of each indicator of cardiovascular disease risk factors under different factors. The multiplicative interaction was also analyzed based on logistic regression models.

#### 3.2.1 Subgroup analysis of PRM intake and risk of glycemic control

**Figure 1:**
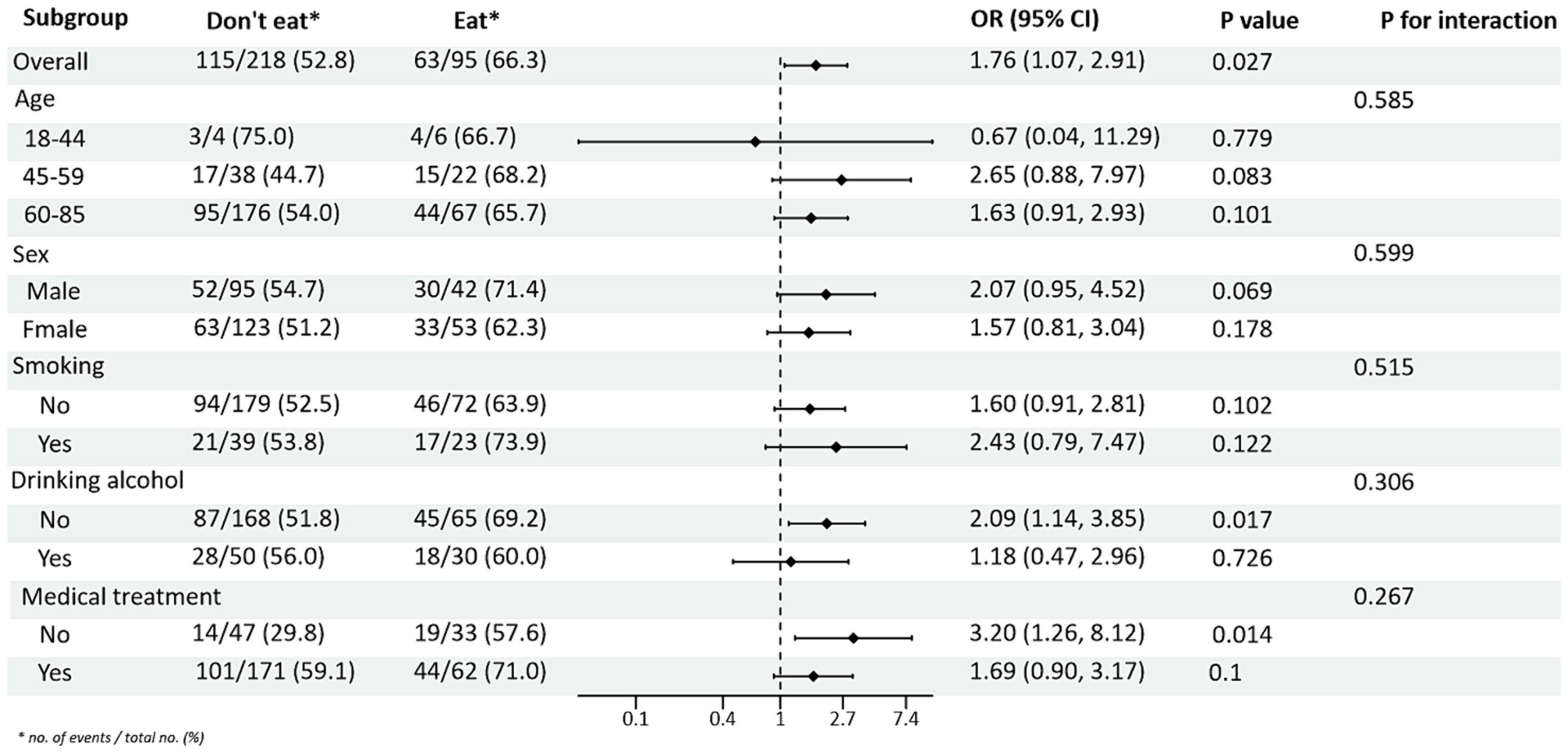
Subgroup analysis of the effects of processed red meat intake or not on glycated hemoglobin A1c (HbA1c).

Subgroup analysis of PRM intake or not and level of glycaemic control showed: In the total population, the risk of non-normal HbA1c index in T2DM patients taking PRMs was 1.76 times higher than that of those not taking PRMs (OR = 1.76; 95% CI 1.07–2.91; *p* = 0.027), suggesting that taking PRMs may increase the outcome event HbA1c control incidence of non-attainment as a risk factor. Across subgroups, the risk of substandard HbA1c control was 2.09 times higher in non-drinkers taking a PRM than in those not taking a PRM (OR = 2.09; 95% CI 1.14–3.85; *p* = 0.017). The risk of substandard HbA1c control was 3.20 times higher in patients without pharmacological treatment who ate PRM than those who did not (OR = 3.20; 95% CI 1.26–8.12; *p* = 0.014; Figure 1). The association between the presence or absence of PRM intake and the risk of FPG control was not statistically different across subgroups (Supplementary Figure 3). In addition, in the multiplicative interaction analysis, there was an effect modifier effect of smoking on the association between PRM intake status and risk of substandard FPG control (Pinteraction = 0.033), i.e., the effect of PRM on the risk of substandard FPG control was influenced by the factor of smoking. No statistically significant interaction terms were seen for any of the other interactions.

#### 3.2.2 Subgroup analysis of PRM intake and risk of blood pressure control

**Figure 2:**
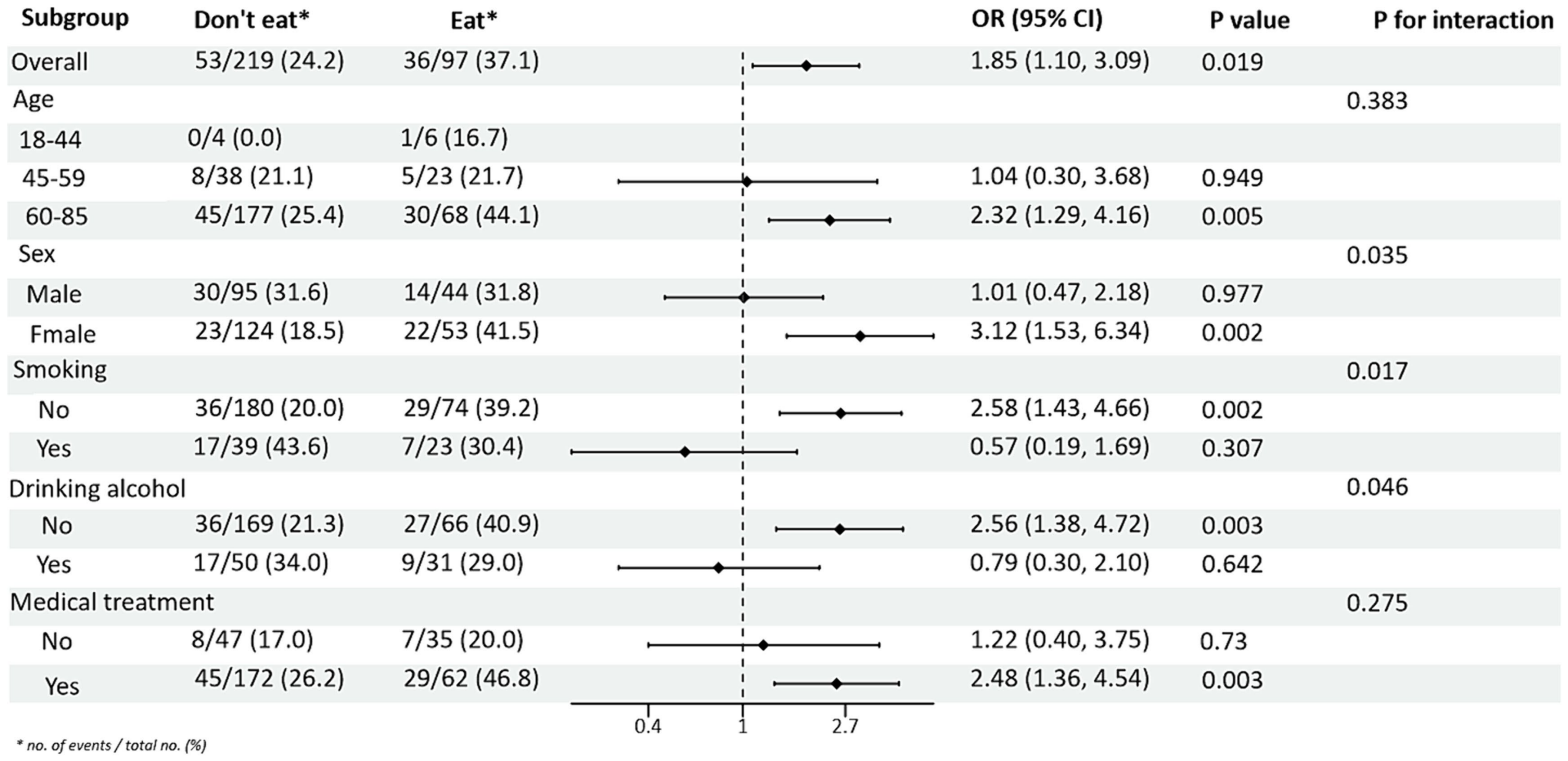
Subgroup analysis of the effects of processed red meat intake or not on systolic blood pressure (SBP).

**Figure 3:**
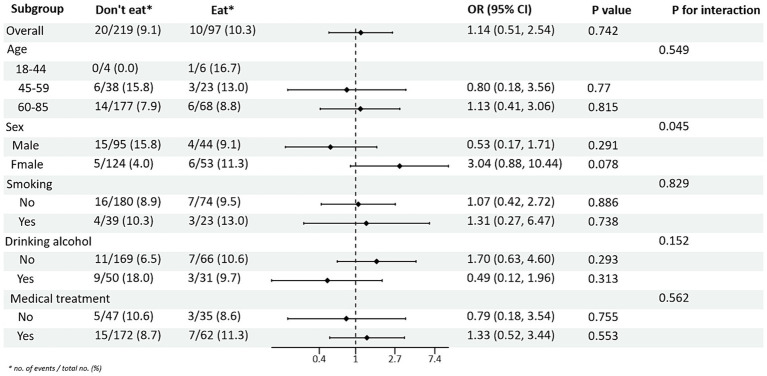
Subgroup analysis of the effects of processed red meat intake or not on diastolic blood pressure (DBP).

Subgroup analysis of PRM intake and blood pressure control showed that in the total population, the risk of abnormal systolic blood pressure index was 1.85 times higher in T2DM patients who ate PRMs than in those who did not (OR = 1.85; 95% CI 1.10–3.09; *p* = 0.019), and no significant difference was seen in diastolic blood pressure. Among the female patients, the risk of abnormal systolic blood pressure index was 3.12 times higher after eating ingested PRM than after not eating PRM (OR = 3.12; 95% CI 1.53–6.38; *p* = 0.002). The risk of abnormal systolic blood pressure indices was 2.58 times higher in non-smokers who ate PRMs than those who did not (OR = 2.58; 95% CI 1.42–4.67; *p* = 0.002). Among patients who did not drink alcohol, the risk of abnormal systolic blood pressure markers was 2.56 times higher with PRM than without (OR = 2.56; 95% CI 1.38–4.73; *p* = 0.003). The risk of an abnormal systolic blood pressure index was 2.48 times higher in patients who chose treatment than in those who did not take PRM (OR = 2.48; 95% CI 1.36–4.55; *p* = 0.003). For diastolic blood pressure, no significant differences were found (*p* > 0.05). In addition, in the multiplicative interaction analyses, there were effect modifiers for sex (Pinteraction = 0.035), smoking status (Pinteraction = 0.017), and alcohol consumption (Pinteraction = 0.046) on the association between PRM intake status and risk of abnormal systolic blood pressure. Sex (Pinteraction = 0.045) had an effect modifier in the association of PRM intake status with the risk of abnormal diastolic blood pressure. That is, the effect of PRM on systolic blood pressure was influenced by sex, smoking, and alcohol consumption factors, and the effect on diastolic blood pressure was influenced by sex factors. No statistically significant interaction terms were seen for any of the other interactions (Figures 2, 3).

#### 3.2.3 Subgroup analysis between PRM intake and lipid levels

**Figure 4:**
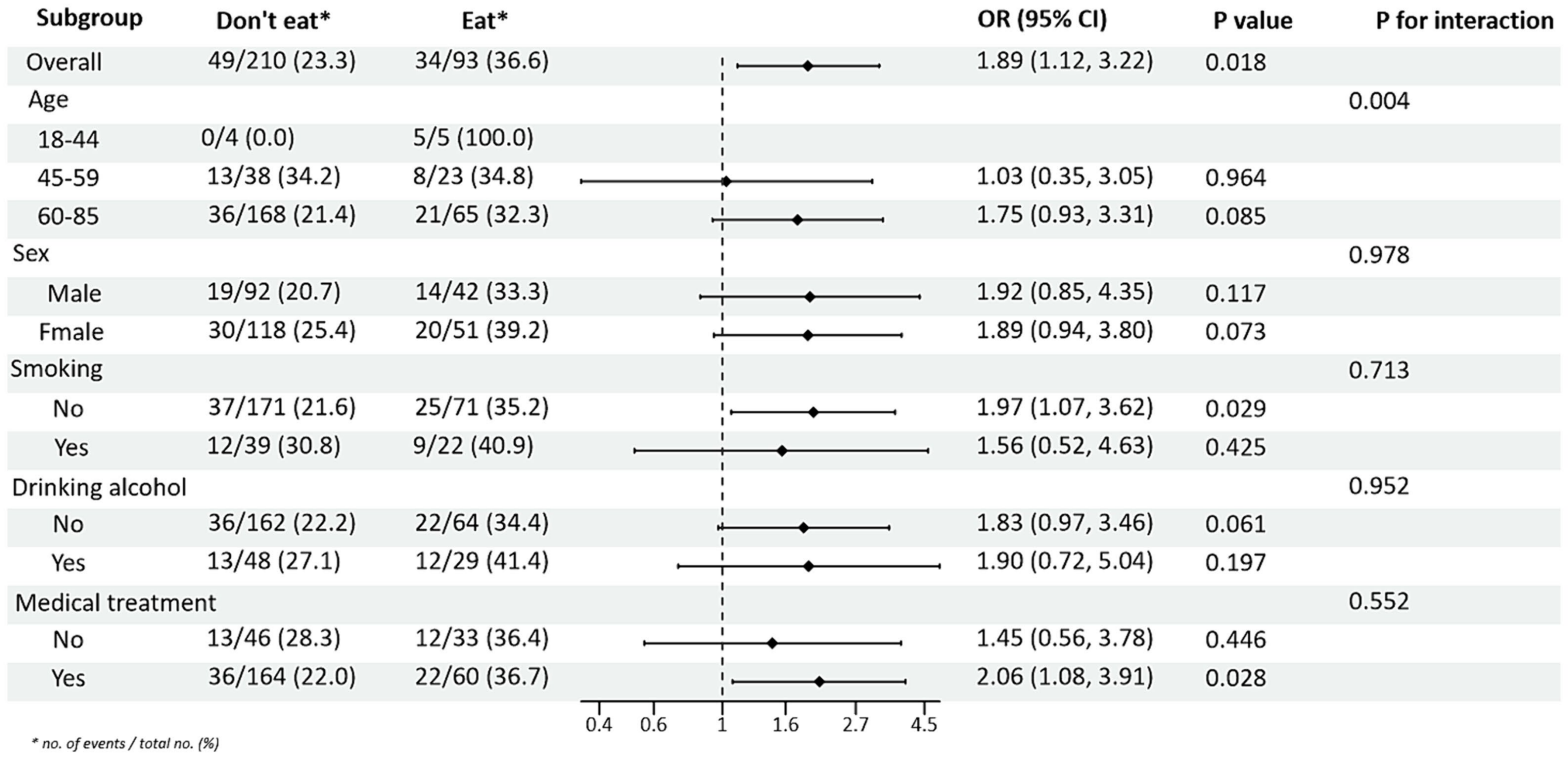
Subgroup analysis of the effects of processed red meat intake on triglycerides (TG).

**Figure 5:**
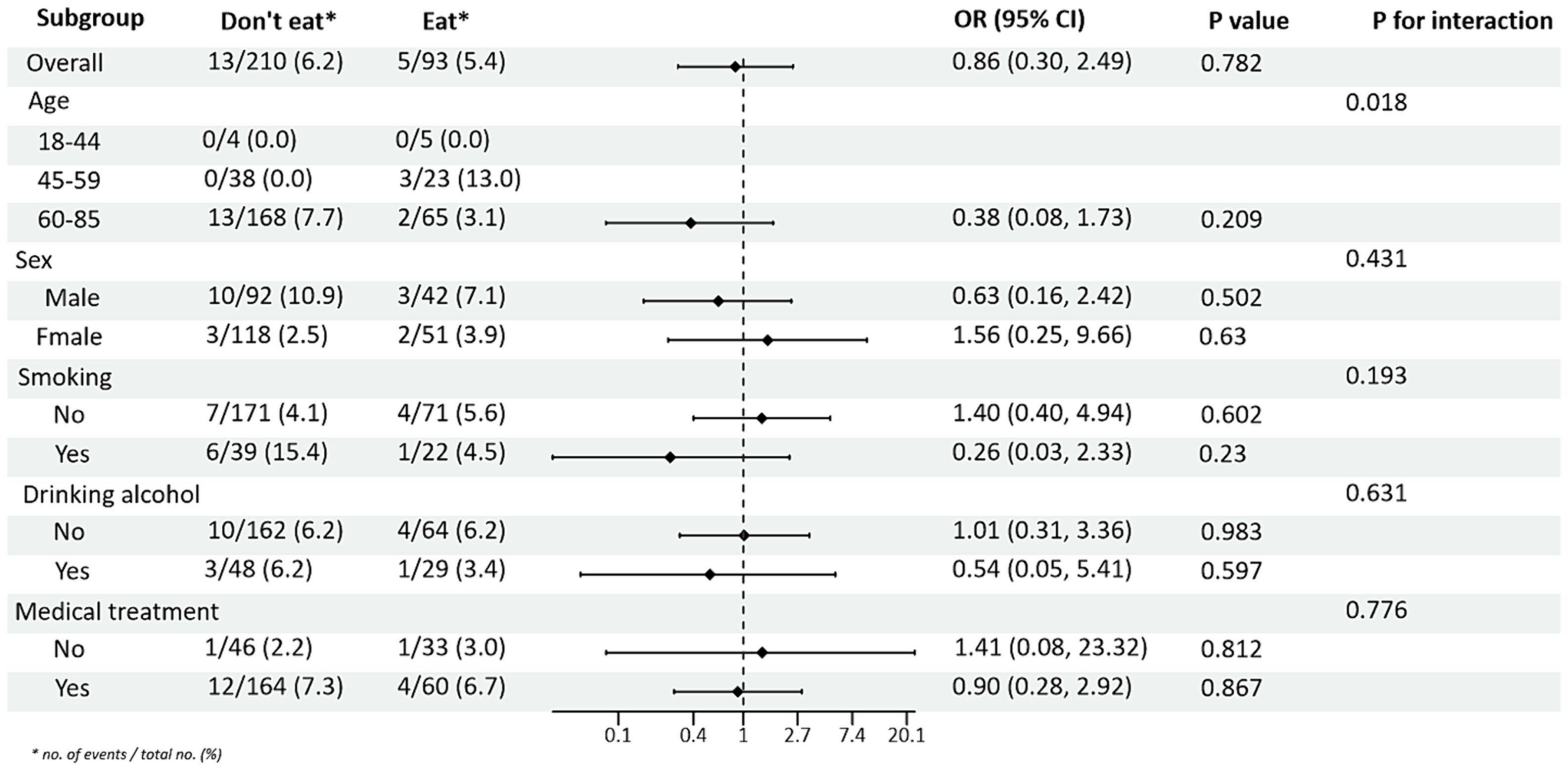
Subgroup analysis of the effect of processed red meat intake on high-density lipoprotein-cholesterol (HDL-C).

Subgroup analyses between PRM intake or not and TG and HDL-C control levels showed (Figures 4, 5): In the total population, PRM intake status was significantly associated with the risk of abnormal TG markers, and the risk of abnormal TG markers in T2DM patients who ate PRM was 1.89 times higher than that of those who did not eat PRM (OR = 1.89; 95% CI 1.12–3.22; *p* = 0.018), and no significant differences were seen in other indicators. It is suggested that eating PRM can increase the risk of abnormal TG levels and may lead to high TG levels. In all subgroups, among non-smoking patients, the risk of abnormal triglyceride indexes was 1.97 times higher by eating PRM than by not eating PRM (OR = 1.97; 95% CI 1.07–3.62; *p* = 0.029). The risk of an abnormal PRM triglyceride index was 2.06 times higher in patients who chose treatment than in those who did not (OR = 2.06; 95% CI 1.08–3.91; *p* = 0.028). In addition, the association of PRM intake or not with the risk of abnormal TC and LDL-C metrics did not reveal relevant differences across subgroups (Supplementary Figures 4, 5). In the multiplicative interaction analyses, there was an effect modifier effect of age on the association of PRM intake status with the risk of abnormal TG metrics (Pinteraction = 0.004) and the risk of abnormal HDL-C metrics (Pinteraction = 0.018). That is, the effect of PRM on TG and HDL-C was influenced by the age factor. No statistically significant interaction terms were seen for TC and LDL-C in the subgroups.

### 3.3 Association of PRM intake with risk of abnormalities in various indicators of cardiovascular disease risk factors

Abnormal levels of control for each of the cardiovascular disease risk factors were used as the outcome variable, and PRM intake was used as the exposure variable. After adjusting for age, sex, smoking, alcohol consumption, medication, and total energy intake, logistic regression models showed that the OR for substandard HbA1c control in patients in the highest PRM intake group, T3 (3.4 ~ 57.2 g/d), was 1.620 times higher than that in the lowest intake, i.e., no intake group, T1 (0.0 ~ 0.0 g/d; OR = 2.620; 95% CI 1.198 ~ 5.732; *p* = 0.016). Whereas the OR for abnormal control of systolic blood pressure levels was 1.025 times higher (OR = 2.025; 95% CI 1.033 ~ 3.968; *p* = 0.040) in patients in the PRM low intake group T2 (0.1 ~ 3.3 g/d) than in the non-intake group T1 (0.0 ~ 0.0 g/d), the OR for substandard control of systolic blood pressure in patients in the highest group T3 (3.4 ~ 57.2 g/d) was 1.166 times higher (OR = 2.166; 95% CI 1.007 ~ 4.660; *p* = 0.048) than that in the no-intake group T1 (0.0 ~ 0.0 g/d). the OR for abnormal TG levels in patients in the highest PRM intake group T3 (3.4 ~ 57.2 g/d) was 1.095 times higher than that in the no-intake group T1 (0.0 ~ 0.0 g/d; OR = 2.095; 95% CI 1.076 ~ 4.078; *p* = 0.030). In addition, for FPG, DBP, TC, LDL-C, and HDL-C, the differences were not statistically significant after adjusting the model (*p* > 0.05; Table 2).

**Table 2:** Association between processed red meat intake and abnormal risk of various cardiovascular risk factors.

Variable	Model 1 OR(95%CI)	*P*	Model 2 OR(95%CI)	*P*
HbA1c				
T1	1.00	–	1.00	–
T2	1.556(0.834–2.903)	0.165	1.857 (0.965 ~ 3.574)	0.064
T3	2.067(1.023–4.175)	0.043	2.620 (1.198 ~ 5.732)	**0.016**
FPG				
T1	1.00	–	1.00	–
T2	0.740(0.395–1.386)	0.346	0.891 (0.465 ~ 1.707)	0.727
T3	1.345(0.621–2.914)	0.453	1.658 (0.715 ~ 3.844)	0.239
SBP				
T1	1.00	–	1.00	–
T2	1.700(0.898–3.219)	0.103	2.025 (1.033 ~ 3.968)	**0.040**
T3	2.048(1.032–4.063)	0.040	2.166 (1.007 ~ 4.660)	**0.048**
DBP				
T1	1.00	–	1.00	–
T2	0.796(0.260–2.433)	0.689	0.862 (0.273 ~ 2.716)	0.799
T3	1.614(0.607–4.288)	0.337	1.028 (0.338 ~ 3.130)	0.961
TC				
T1	1.00	–	1.00	–
T2	0.990(0.269–3.642)	0.988	0.868 (0.223 ~ 3.387)	0.839
T3	2.912(1.024–8.282)	0.045	1.860 (0.525 ~ 6.590)	0.336
TG				
T1	1.00	–	1.00	–
T2	1.991(1.049–3.780)	0.035	2.095 (1.076 ~ 4.078)	**0.030**
T3	1.769(0.858–3.650)	0.123	1.410 (0.631 ~ 3.147)	0.402
HDL-C				
T1	1.00	–	1.00	–
T2	1.237(0.386–3.960)	0.720	2.265 (0.633 ~ 8.106)	0.209
T3	0.389(0.049–3.057)	0.369	0.341 (0.039 ~ 2.984)	0.331
LDL-C				
T1	1.00	–	1.00	–
T2	0.990(0.108–9.049)	0.993	0.750 (0.074 ~ 7.607)	0.807
T3	4.176(0.898–19.425)	0.068	2.835 (0.436 ~ 18.441)	0.275

Model 1: model without any variable adjustment; Model 2: Adjusted for age, sex, smoking, alcohol consumption, drug treatment, and total energy intake. Full name and abbreviation: glycated hemoglobin A1c (HbA1c), fasting plasma glucose (FPG), systolic blood pressure (SBP), diastolic blood pressure (DBP), triglycerides (TG), total cholesterol (TC), high-density lipoprotein-cholesterol (HDL-C) and low-density lipoprotein-cholesterol (LDL-C).

### 3.4 Dose–response relationship between processed red meat intake and glycemic control

**Figure 6:**
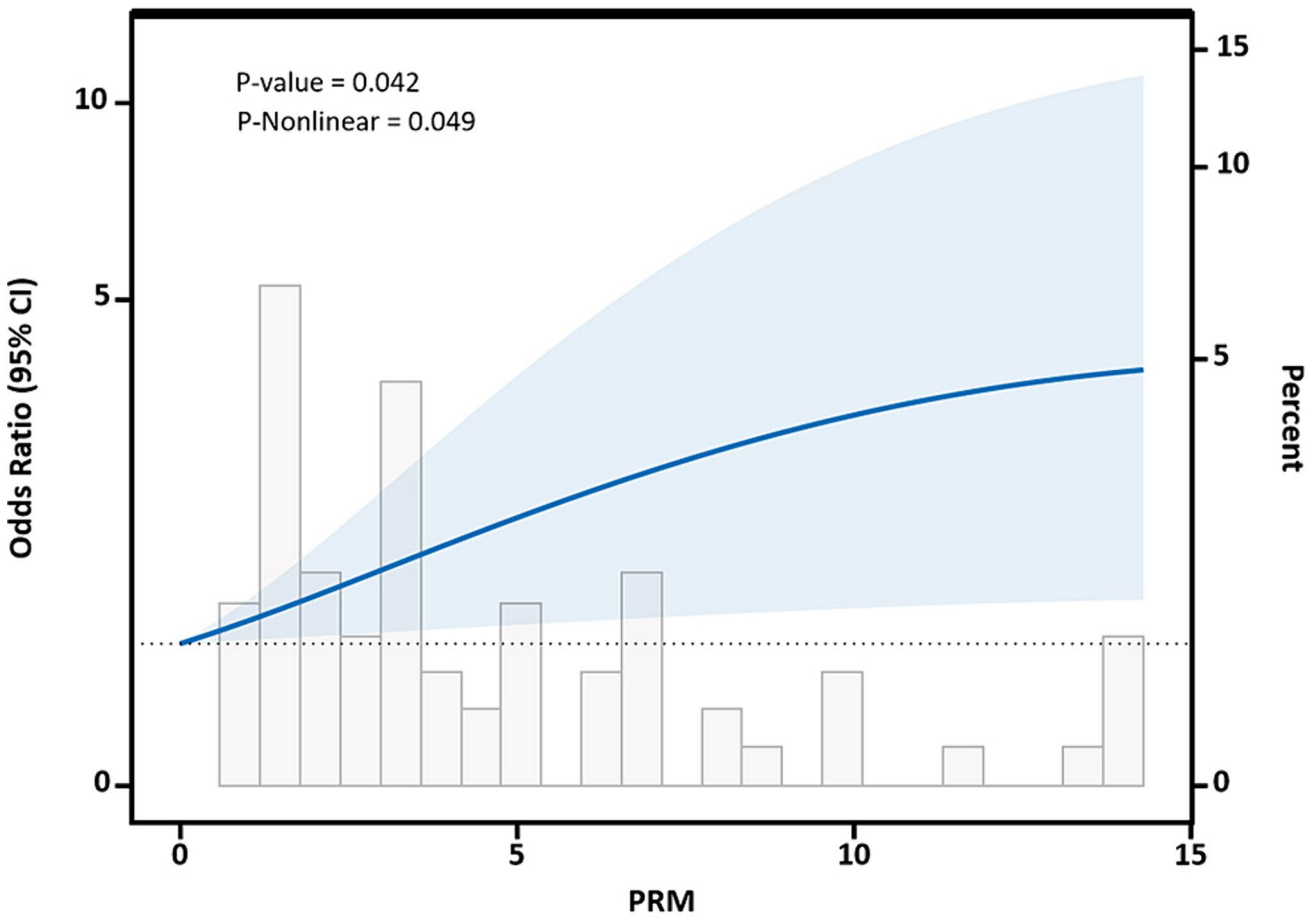
Dose–response relationship between processed red meat(PRM) intake and glycated hemoglobin A1c (HbA1c) control levels. Figure shows a restricted cubic spline model of processed red meat intakes to the HbA1c odds ratio. HbA1c was adjusted for age, sex, smoking, alcohol consumption, medication, and total energy intake. The shaded portion represents the 95% confidence interval.

Restricted cubic spline curves based on Logistic regression and linear regression were used to analyze the dose–response relationship between PRM intake and the levels of cardiovascular disease risk factors. The results of restricted cubic spline plots between PRM intake and level of glycemic control in T2DM patients are shown in the figure. After adjusting for potential confounding factors, there was a non-linear dose–response relationship between PRM intake and the risk of poor HbA1c control in T2DM patients (*p* < 0.05, P non-linear <0.05). The figure shows that OR increased steadily until PRM intake was about 10 g/d, and the rate of increase in OR slowed down after 10 g/d (Figure 6). As PRM intake increased, the overall risk of substandard FPG control tended to increase and then decrease, but no statistically significant associations were observed (*p* > 0.05; Supplementary Figure 6).

### 3.5 Dose–response relationship between processed red meat intake and blood pressure control

**Figure 7:**
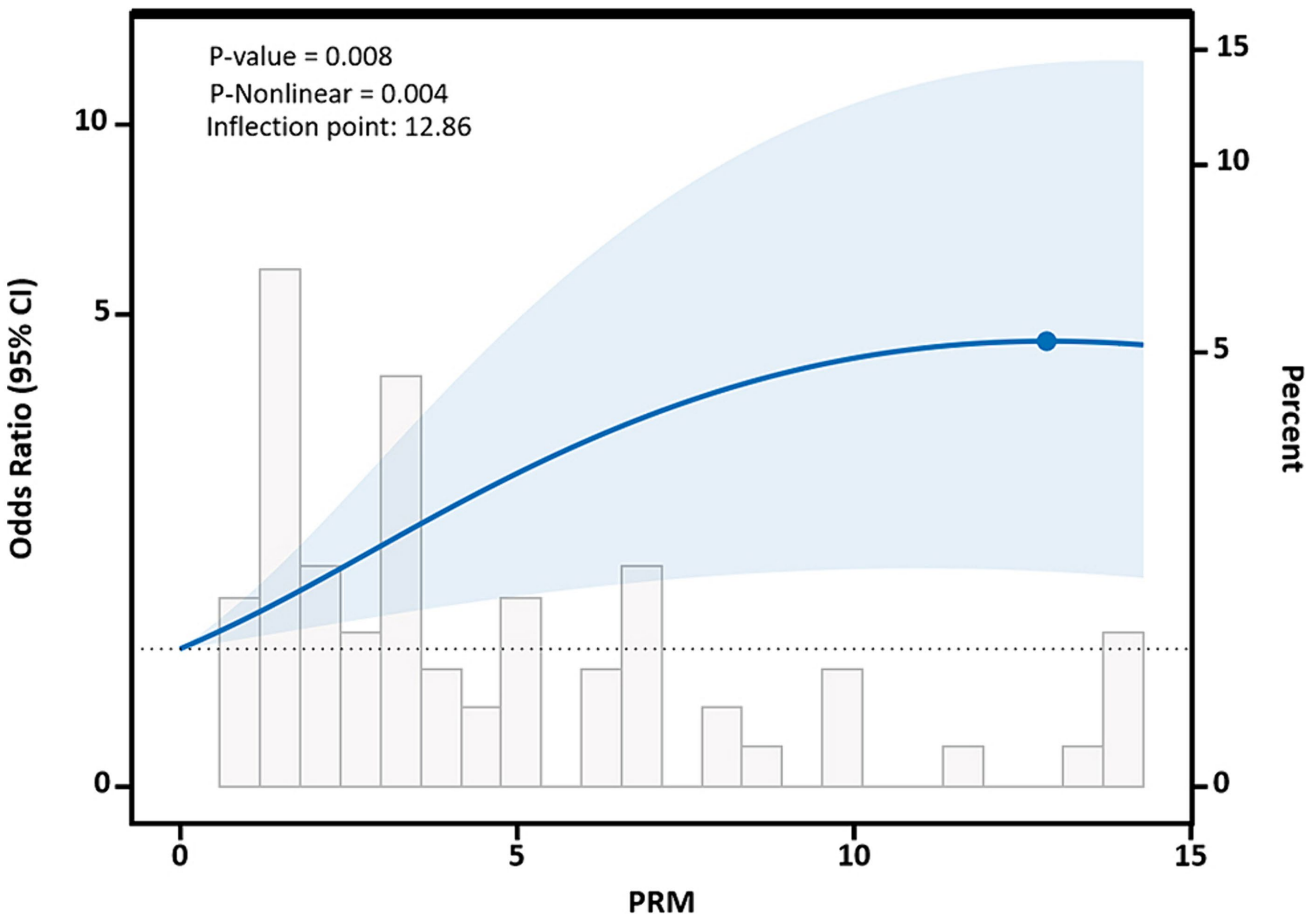
Dose–response relationship between processed red meat(PRM) intake and systolic blood pressure (SBP) indicators. Figure shows a restricted cubic spline model of processed red meat intakes to SBP odds ratios. They are adjusted for age, sex, smoking, alcohol consumption, medication, and total energy intake. The shaded portion represents the 95% confidence interval.

After adjusting for potential confounding factors, restricted cubic spline results showed that there was a nonlinear dose–response relationship between PRM intake and the risk of abnormal systolic blood pressure control in T2DM patients (*p* < 0.05, P nonlinear = 0.004), which was similar to an “L curve,” and PRM at peak systolic blood pressure was 12.86 g/d. Figure shows, when the PRM intake was more than 0.0 g/d, OR obvious rise, when the PRM intake to about 10 g/d, systolic blood pressure increased risk of abnormal speed slow, until the PRM intake peaked at 12.86 g/d, and then began to decline, as with the increase of the PRM intake, increased risk of abnormal systolic blood pressure, When the patient’s daily PRM intake reached 12.86 g, the risk began to slowly decrease (Figure 7). With the increase of the PRM intake, DBP control falls below the risk of a rising trend on the whole, but it was not observed the PRM was a statistically significant association between intake and DBP (*p* > 0.05; Supplementary Figure 7).

### 3.6 Dose–response relationship between processed red meat intake and lipid levels

**Figure 8:**
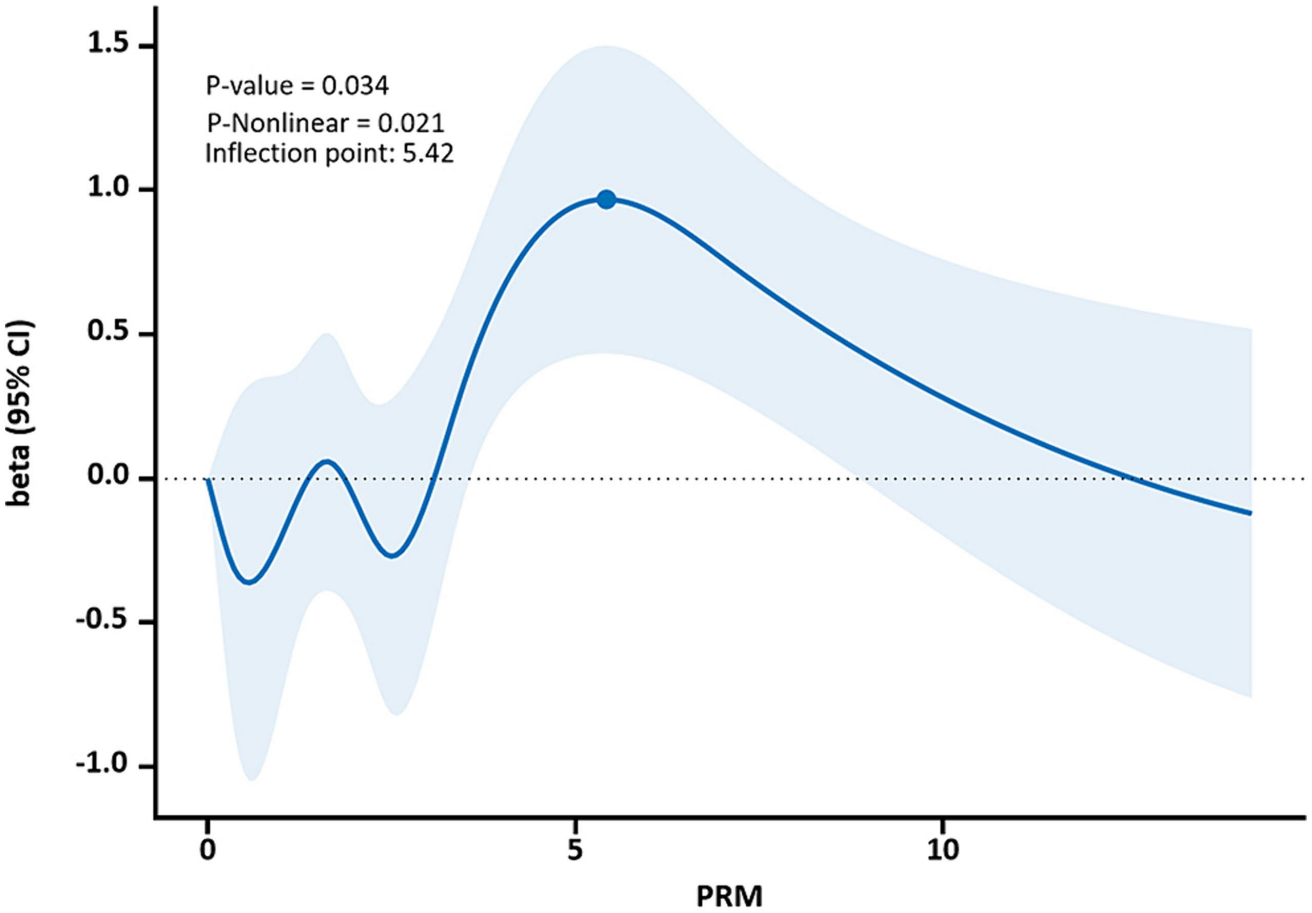
Dose–response relationship between processed red meat(PRM) intake and total cholesterol (TC) indicators. Figure shows a restricted cubic spline model of processed red meat intakes to TC odds ratios. They are adjusted for age, sex, smoking, alcohol consumption, medication, and total energy intake. The shaded portion represents the 95% confidence interval.

**Figure 9:**
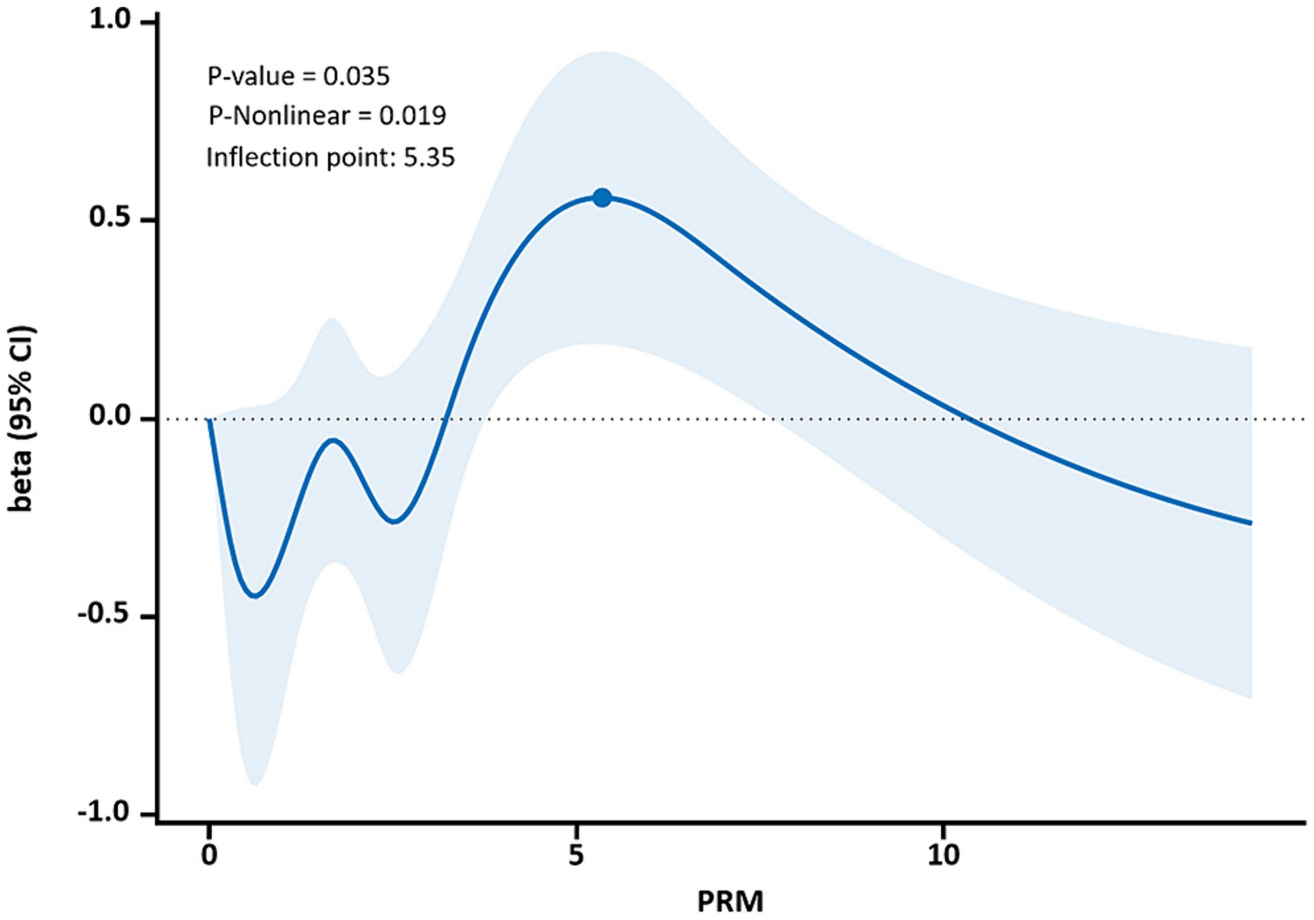
Dose–response relationship between processed red meat(PRM) intake and low-density lipoprotein-cholesterol (LDL-C) indicators. Figure shows a restricted cubic spline model of processed red meat intakes to LDL-C odds ratio. They are adjusted for age, sex, smoking, alcohol consumption, medication, and total energy intake. The shaded portion represents the 95% confidence interval.

The restricted cubic spline results showed a non-linear dose–response relationship between PRM intake and TC and LDL-C concentrations in T2DM patients (*p* < 0.05, P non-linear <0.05). The two were similar to an “inverted U curve”; the PRM of TC was 5.42 g/d, and the PRM of LDL-C was 5.35 g/d at the peak. TC levels increased with PRM intake until about 5.42 g/d and then began to decrease (Figure 8). The level of LDL-C increased with PRM intake until about 5.35 g/d and then began to decline (Figure 9). With the increase in PRM intake, the levels of TG and HDL-C increased first and then decreased, but no statistically significant association was observed (*p* > 0.05; Supplementary Figures 8, 9).

## 4 Discussion

The management of cardiovascular disease risk factors in patients with T2DM, especially the control of blood pressure, lipids, and glucose metabolism abnormalities, has become one of the most urgent problems in the current public health field. A major study (28) found that only a minority, less than one-twelfth, of the Chinese adult population aged 35–75 years, had achieved standard blood pressure control. Another survey revealed that 18 and older in patients with T2DM, and only 23.0% achieved enough blood sugar control target (< 7.0 tendency for L, fasting glucose HbA1c < 7.0%) (29). This low control rate not only poses serious physical and mental health challenges for patients but also highlights the importance of dietary habits in diabetes management (30, 31). Studies have shown that red meat is an important component of total dietary intake in many populations, and its role as one of the major foods may play an important role in the development of T2DM and CVD complications. Numerous meta-analyses have also confirmed the significant association between processed meat intake and increased risk of diabetes (32) and cardiovascular disease (33) in Western populations. *Per capita*, PRM intake is low in Chinese residents (5.0 g/d vs. 17.0 g/d; global *per capita* PRM intake in 2018) (34), and it is noteworthy that meat consumption in developing countries is increasing at a rate of 5 to 6% (35), with red meat accounting for the largest share. Despite the low *per capita* intake of PRM in China, the recommended intake regarding PRM is not explicitly mentioned in existing guidelines. Meanwhile, the association between PRM and CVD risk factors in T2DM patients remains unclear. Therefore, it is important to study the association between PRM and CVD risk factors such as blood pressure, lipid, and glucose metabolism abnormalities in T2DM patients to optimize tertiary prevention and reduce the risk of CVD in T2DM patients. Based on this, this study analyzed cross-sectional data of 316 T2DM patients in a community in Bengbu City.

In this study population, the mean intake of PRM was 2.03 g/d, which was lower than that reported in the 2018 Global and Chinese Nutrition and Health Surveys. The mean daily intake of PRM in this population was lower than that of European countries such as Ireland (36) and Denmark (37) and American countries such as the United States (PRM: 44.5 g/d), Mexico (PRM: 40.0 g/d), and Canada (PRM: 41.8 g/d) (38), but similar to that of Asian countries such as South Korea (PRM: 5.9–7.2 g/d) (39, 40), which are all low intake of PRM. All of them belong to the population with a low intake of PRM. The present study showed that T2DM patients in the high intake group T3 (3.4–57.2 g/d) were more likely to be male, current smokers and alcohol drinkers compared to those in the T1 group (0.0–0.0 g/d); patients in the T3 group had a relatively younger mean age and higher total energy intake. This may be because males and younger patients were more likely to attend social gatherings, order take-out and eat out more frequently, as well as having a higher frequency of dietary behaviors such as smoking, alcohol consumption, and consumption of processed red meats, beverages, and pastries and snacks, and to be more physically inactive, with lower levels of physical activity, and were more likely to adopt unhealthy dietary patterns (based on refined grains, red and processed meats, high-fat dairy products, sugary drinks and sweets, and fried foods), as well as to have higher energy intake (beverages and sweets, and fried foods) (41–43). In addition, the results of this study also showed that PRM intake was higher in patients on medication, which may be because these patients were not concerned with knowledge about glycemic control, perceived their level of glycemic control to be good, and believed that small amounts of PRM intake may not hurt glycemic control.

In the present study, the mean daily intake of PRM in this population was found to be 2.03 g/d, with only 30.7% of the participants consuming PRM, and their mean intake was 6.6 g/d. At this particular level, our study revealed a significant association between PRM intake status and the prevalence of substandard HbA1c, systolic blood pressure, and triglyceride control. However, for total cholesterol, HDL-C, and LDL-C control levels, we did not observe statistically significant associations between PRM intake status and these metrics in different subgroups. This result may be attributed to the non-uniformity of judgment criteria for PRM definition and individual differences among patients, and the specific mechanism of action deserves further in-depth investigation in the future. We observed a significant interaction between smoking and PRM intake on the risk of substandard FPG control. After adjusting for possible confounders, the logistic regression model showed that the OR for substandard HbA1c control was 1.620 times higher in patients in the highest PRM intake group, T3 (3.4–57.2 g/d), than in the lowest intake, i.e., non-intake group, T1 (0.0–0.0 g/d). The dose–response relationship showed that the risk of substandard HbA1c control began to rise significantly as soon as PRM intake was initiated, suggesting that even very small amounts of PRM intake may adversely affect glycemic control. It is also the “gold standard” of glycemic control, suggesting that significant changes in glycemic control have begun to occur at current levels of PRM intake. Our study did not find an association between PRM intake and the risk of suboptimal FPG control, possibly because fasting glucose varies with diet, exercise, etc., and responds to immediate glucose levels. However, this is in contrast to the findings of Amanda M Fretts et al. in Caucasians (44), where the meta-analysis indicated that PRM was associated with higher FPG concentrations, with each additional 50 g of PRM per day being associated with a 0.021 mmol/L increase in FPG. The difference between this and our findings may be related to ethnicity, or it may be attributed to the overall lower intake of patients with T2DM compared to Caucasians. Although no previous studies have directly examined the dose–response relationship between PRM intake and glycemic control, it has been reported in the literature (45, 46) that there is a linear dose–response relationship between PRM intake and the risk of T2DM in adults, with a 27% increase in the risk of T2DM for every 20 g/d increase in PRM. This is similar to the results of the present study. A study by Yu H also noted that consumption of 50 g PRM per day was associated with a 51% increased risk of T2DM (32). This is also similar to the results of the present study. In contrast, two cross-sections in Korea (mean intake of 5.3 g/d in the high intake group) and Japan (mean intake of 13.5 g/d in the high intake group) did not find a significant effect of PRM intake on the risk of T2DM (39, 47), and this discrepancy may stem from the fact that the present population was a population of patients with T2DM with long-term metabolic disorders. Processed red meat intake levels that do not adversely affect healthy populations do not apply to patients with T2DM, and only very low levels of intake do not have an effect. Therefore, the present study suggests a reduction in processed red meat intake in patients with T2DM.

Despite the low average intake of PRMs (2.0 g/d), they still produce adverse effects, probably due to the addition of more saturated fats, sodium, sugars, nitrates, and nitrites, and the production of higher amounts of advanced glycosylation end products (AGEs) and heterocyclic amines during high-temperature processing. Excess saturated fat may increase the risk of central obesity, cause ectopic deposition of serum-free fatty acids, inhibit insulin signaling, lead to insulin resistance, and elevate blood glucose levels (48). Secondly, the sodium content per gram of PRM is about 4-fold higher than that of URM, and high sodium intake increases the risk of hypertension, which is often accompanied by insulin resistance and T2DM and is one of the independent risk factors for T2DM (49, 50). In addition, excessive added sugars in PRMs produce fructose after digestion and absorption, which can lead to an increase in liver *de novo* fat, and when the liver cannot compensate for these increased lipids, insulin resistance is triggered, causing blood glucose levels to rise (51, 52). In contrast, PRM containing high levels of nitrates and nitrites may lead to the formation of N-nitroso compounds, which can result in DNA damage, lipid peroxidation, and inflammation, with toxic effects on pancreatic β-cells, a decrease in insulin secretion, and a high level of blood glucose levels (53). In addition, AGEs and heterocyclic amines formed during high-temperature preparation and processing of PRMs lead to elevated levels of hydroxyl radicals, oxidative stress, and inflammation, resulting in insulin resistance and ultimately high blood glucose levels over a long time (54). This is similar to the results of a previous study (55); this study also showed that BMI was the main mediator of the level of glycemic control by PRM, but this result was not found in this study.

This study also found that sex, smoking, and alcohol consumption showed effect modifying effects on the associations between PRM intake and risk of SBP control and that there was an effect modifying effect of sex on the associations between PRM intake status and risk of DBP abnormality. That is, the effect of PRM on DBP was influenced by the sex factor. In addition, there was an effect modifier effect of age on the association of PRM intake status with the risk of abnormal TG metrics and the risk of abnormal HDL-C metrics. Specifically, the association between PRM intake and the risk of abnormal BP control was more significant in the female population than in the male population. Logistic regression modeling showed that the OR for abnormal SBP level control was 1.025 times higher in the low PRM intake group of T2 (0.1–3.3 g/d) than in the no intake group of T1 (0.0–0.0 g/d), and that systolic BP control was not as high in the highest group of T3 (3.4–57.2 g/d) patients with substandard SBP control had an OR 1.166 times higher than that of the non-intake group T1 (0.0 to 0.0 g/d). The OR for abnormal TG levels in patients in the highest group was 1.095 times higher than in the non-intake group. In addition, after correcting for confounders, for FPG, DBP, TC, LDL-C, and HDL-C, the differences were not statistically significant. In this regard, a stratified analysis by Linda M. OUDE GRIEP et al. showed a significant direct association between PRM and SBP in Western women (56), which is similar to our findings. It further suggests the importance of PRM intake control in BP management. In addition, PRM consumption significantly increased the risk of abnormal SBP control in nonsmoking T2DM patients compared with current smokers, and the association between PRM intake and the risk of substandard SBP control was also more pronounced in patients who did not currently consume alcohol compared with those who did. This result may be limited by the fact that the present study was a small sample investigation. The dose–response relationship showed an approximate “L-curve” relationship between PRM intake and the risk of suboptimal control of SBP in T2DM patients, with the OR rising significantly when PRM intake exceeded 0.0 g/d until about 10 g/d when the increase in the risk of SBP abnormality slowed down to a peak of 12.86 g/d and then began to decline. and then began to decline. On the other hand, there was an “inverted U” relationship between PRM intake and TC and LDL-C concentration levels. This suggests that although the *per capita* intake of PRMs in our community is relatively low, it still affects blood pressure and lipid levels and that patients with T2DM should reduce their intake of PRMs, especially in this community, where the average intake of PRMs was 6.6 g/d among those who chose to eat PRMs.

This may be attributed to the fact that PRMs contain too many additives, such as a high percentage of saturated fats, added nitrates, and nitrites (49, 50), which contribute to the elevation of blood pressure. It is shown that the current PRM intake adversely affects the lipid profile levels of patients. However, Hassannejad et al.’s (57) study showed that each additional serving of PRM intake was not significantly associated with the levels of lipid profile (including TC, TG, HDL-C, and LDL-C) in a group of the healthy Iranian population. A meta-analysis by Dena Zeraatkar et al. (58) also supported Hassannejad R and found that a reduction of three servings per week of PRM intake was associated with a small reduction in the risk of cardiovascular death, stroke, and T2DM. That is, the degree of association between PRM intake and adverse cardiometabolic outcomes was very small. This may be due to the very low consumption of PRMs and the fact that the study was conducted in healthy adults, our study population being patients with T2DM. It has also been suggested that PRM is associated with an increased risk of CVD. The results of a dose–response analysis by Chen G-C et al. (59) showed that 50 g/d PRM intake was significantly associated with an 11% increase in the risk of CVD in adults, which is similar to the results of the present study. In addition, fat accumulation figured prominently in the association between red meat consumption and insulin resistance and inflammation (48). Parvin Mirmiran (60) et al. also noted a significant effect of saturated fat intake on TC levels. This is also similar to our findings. In addition, Lajous M et al. found (61) that PRM intake of ≥250 g/w (35.7 g/d) significantly increased the risk of hypertension by 17% compared with 50 g/w (7.1 g/d), a finding that also supports the findings of the present study. In addition, the risk of abnormal SBP levels was significantly higher in the PRM intake T2 and T3 groups than in the T1 group, probably due to the addition of nitrates and nitrites to PRMs that contribute to the elevation of blood pressure (53). Some studies have shown that the sodium content per gram of PRM is about four times higher than that of URM, and excessive sodium intake activates the renin-angiotensin (RAS) system, which increases the production of angiotensin-II, promotes inflammatory responses, increases peripheral vascular resistance, impairs arterial vascular compliance, and leads to elevated blood pressure, increased pulse pressure, and the development of hypertension (49, 50). Together, these studies emphasize the impact of PRM intake on cardiovascular health in T2DM patients, suggesting that we need to pay more careful attention to and control PRM intake.

In summary, T2DM patients should be more vigilant about PRM intake in their diet. Such foods may lead to abnormalities in blood pressure, blood lipids, and glucose metabolism, which in turn exacerbate diabetic pain and increase the risk of CVD complications. Emphasis on controlling blood glucose, blood pressure, and lipid levels becomes one of the important therapeutic measures to reduce diabetic pain and prevent the management of CVD complications. Therefore, it is recommended that in the tertiary prevention of diabetes and CVD prevention, patients with T2DM, especially those who are younger and not taking medication, should reduce the intake of PRMs. Promoting education on healthy eating and adopting a healthier diet, such as increasing the intake of vegetables and fruits, whole grains, and regular visits to the community to test the levels of cardiovascular disease risk factor indicators, and making timely adjustments to reduce the risk of CVD in patients with diabetes. These recommendations can guide clinical practice and public health policy and can help improve the overall health of people with T2DM.

The significant strength of this study lies in the finding that a small intake of PRM significantly increases the rate of substandard glycemic and systolic blood pressure control as well as increases lipid levels in patients with T2DM. It analyzed the specific effects of PRM intake on cardiovascular disease risk factors such as blood pressure, lipids, and abnormal glucose metabolism in patients with T2DM and explored the dose–response relationship between PRM intake and the concentrations of various indices of cardiovascular metabolism. To improve the reliability of our results, we adjusted for several potential confounders in the restricted triple sample strips, and this approach allowed us to meticulously assess the effects of PRM intake on the associated risks in a continuous distribution. However, there are some limitations of this study that need to be explicitly noted here: (1) This is a small-sample cross-sectional study with limitations due to the small sample size of T2DM in the community-specific disease database, which may have some impact on the results. Due to the limitation of population size and the fact that only one-third of the population consumes PRM, its findings need to be taken with caution when generalizing to other populations. (2) The study sample was recruited only in Bengbu, China, and may not be fully representative of the entire general population, which may limit the generalizability of our findings. (3) Due to the characteristics of observational studies and the complex relationship between PRM, T2DM, and CVD risk factors, confounding factors may not have been adequately identified and controlled. (4) Patients’ dietary intake relied on self-reporting and was collected using a semiquantitative food frequency questionnaire combined with food molds, and there were problems with the inherent lack of precision in the data collected by the FFQ as well as sampling blood samples only once, which may have been subject to recall bias and reporting bias, and limitations in capturing PRM portion sizes and dietary intake with greater precision. (5) Future studies could establish cohort or case–control studies to observe the effects of long-term changes in PRM intake on the level of glycemic, blood pressure, and lipid control, and to explore differences in the relationship of PRM intake to glycemia, blood pressure, and lipids between patients with T2DM and non-T2DM populations. (6) Consider repeated dietary measurements and repeated analyses of blood samples in the study population, which could provide deeper insights through repeated analyses. (7) With the small number of T2DM patients consuming PRM in the total sample size of this study, linear regression of individual subject data in the total population consuming PRM could be considered in the future to explore some of the potential associations in a more sensitive manner.

## 5 Conclusion

The present study found a positive association between processed red meat intake and cardiovascular disease risk factors, with a small intake of PRMs significantly increasing the rate of substandard glycemic and systolic blood pressure control as well as increasing lipid levels in patients with T2DM. Current PRM intake is low but has begun to adversely affect glycemic, blood pressure, and lipid profiles. This finding suggests that limiting PRM intake in the management of T2DM may help to reduce the risk of developing CVD and has implications for clinical practice. It also adds to the growing body of evidence on the role of PRMs in several diet-related NCDs, suggests that dietary guidelines should continue to emphasize dietary patterns low in red and processed meats, and helps to inform the development of specific recommended intakes for PRMs as well as public policies for diabetes and CVD prevention and management.

## Data Availability

The raw data supporting the conclusions of this article will be made available by the authors, without undue reservation.

## Supplementary material

SUPPLEMENTARY FIGURE 1 Box plot of processed red meat(PRM) intake.

SUPPLEMENTARY FIGURE 2 The processed red meat intake of the probability density histogram.

SUPPLEMENTARY FIGURE 3 Subgroup analysis of the effects of processed red meat intake or not on fasting plasma glucose (FPG).

SUPPLEMENTARY FIGURE 4 Subgroup analysis of the effect of processed red meat intake on total cholesterol (TC).

SUPPLEMENTARY FIGURE 5 Subgroup analysis of the effect of processed red meat intake on low-density lipoprotein-cholesterol (LDL-C).

SUPPLEMENTARY FIGURE 6 Dose-response relationship between processed red meat(PRM) intake and fasting plasma glucose (FPG) control level. Figure shows a restricted cubic spline model of processed red meat intakes to FPG odds ratio. FPG was adjusted for age, sex, smoking, alcohol consumption, medication, and total energy intake. The shaded portion represents the 95% confidence interval.

SUPPLEMENTARY FIGURE 7 Dose-response relationship between processed red meat(PRM) intake and diastolic blood pressure (DBP) indicators. Figure shows a restricted cubic spline model of processed red meat intakes to DBP odds ratio. They are adjusted for age, sex, smoking, alcohol consumption, medication, and total energy intake. The shaded portion represents the 95% confidence interval.

SUPPLEMENTARY FIGURE 8 Dose-response relationship between processed red meat(PRM) intake and triglycerides (TG) indicators. Figure shows a restricted cubic spline model of processed red meat intakes to TG odds ratios. They are adjusted for age, sex, smoking, alcohol consumption, medication, and total energy intake. The shaded portion represents the 95% confidence interval.

SUPPLEMENTARY FIGURE 9 Dose-response relationship between processed red meat(PRM) intake and high-density lipoprotein-cholesterol (HDL-C) indicators. Figure shows a restricted cubic spline model of processed red meat intakes to HDL-C odds ratio. They are adjusted for age, sex, smoking, alcohol consumption, medication, and total energy intake. The shaded portion represents the 95% confidence interval.
