# Supplementary material for: Association between processed red meat intake and cardiovascular risk factors in patients with type 2 diabetes: a cross-sectional study from China
Source: Front Nutr. 2024 Aug 29;11:1438953. doi: 10.3389/fnut.2024.1438953 (PMC11390537; doi:10.3389/fnut.2024.1438953)
Supplement: Supplementary file 1 [file Table_1.DOCX]

Supplementary table 1. The processed red meat intake

| **Categories** | **Groups of intake(N)** | **Processed red Meat intake (g/d)** |
| --- | --- | --- |
| **Mean** |  | 2.03 |
|  | No(219**)** | 0.00 |
|  | Yes(97**)** | 6.60 |
| **Median** |  |  |
|  | No(219**)** | 0.00 |
|  | Yes(97**)** | 3.30 |
